# Keratin 19 (Krt19) is a novel marker gene for epicardial cells

**DOI:** 10.3389/fgene.2024.1385867

**Published:** 2024-05-20

**Authors:** Juan Xu, Yiting Deng, Guang Li

**Affiliations:** Department of Cell Biology, Center for Integrative Organ Systems, University of Pittsburgh School of Medicine, Pittsburgh, PA, United States

**Keywords:** KRT19, epicardial cell, DTA, ablation, lineage tracing, WT1

## Abstract

Epicardial cells regulate heart growth by secreting numerous growth factors and undergoing lineage specification into other cardiac lineages. However, the lack of specific marker genes for epicardial cells has hindered the understanding of this cell type in heart development. Through the analysis of a cardiac single cell mRNA sequencing dataset, we identified a novel epicardial gene named *Keratin 19* (*Krt19*). Further analysis of the expression patterns of *Krt19* and *Wt1*, a well-known epicardial gene, revealed their preferences in major cardiac cell types. Using lineage-tracing analysis, we analyzed *Krt19-CreER* labeled cells at multiple time windows and found that it labels epicardial cells at both embryonic and neonatal stages. Furthermore, we studied the function of epicardial cells using a diphtheria toxin A chain (DTA)-based cell ablation system. We discovered that *Krt19-CreER* labeled cells are essential for fetal heart development. Finally, we investigated the function of *Krt19-CreER* and *Wt1-CreER* labeled cells in neonatal mouse development. We observed that the *Krt19-CreER*; *Rosa-DTA* mice displayed a smaller size after tamoxifen treatment, suggesting the potential importance of *Krt19-CreER* labeled cells in neonatal mouse development. Additionally, we found that *Wt1-CreER*; *Rosa-DTA* mice died at early stages, likely due to defects in the kidney and spleen. In summary, we have identified *Krt19* as a new epicardial cell marker gene and further explored the function of epicardial cells using the *Krt19-CreER* and *Wt1-CreER*-mediated DTA ablation system.

## Introduction

Heart development is an essential embryonic process that involves multiple cell lineages and is tightly regulated at the cellular and molecular levels. If this process goes awry, it will lead to congenital heart diseases (CHDs), which account for a significant portion of stillbirths and is present in 1%–2% of all live births (Triedman and Newburger, 2016). The epicardium, a membranous layer covering the outside of the myocardium, not only acts as a pool of multi-potential progenitor cells contributing to the development of fibroblasts and smooth muscle cells but also acts as an important source of mitogenic signals to maintain the continued growth and differentiation of the heart (von et al., 2011; von Gise and Pu, 2012; Riley, 2012; Simoes and Riley, 2018). A systematic analysis of epicardial cell function is critical not only for understanding these normal heart development processes but is also required for understanding the molecular mechanisms of CHDs such as Left Ventricular Non-Compaction Cardiomyopathy (Zhang et al., 2013; Villa et al., 2016; Jang et al., 2022).

Epicardial cells are known to develop from the proepicardial organ (PEO) and highly express many genes, including *Wt1* (Cao et al., 2020). *Wt1* is a transcription factor, mutations of which in mice lead to death at early stages before E14.5^2^. The *Wt1-CreER* mouse line has been utilized to trace the lineage descendants of epicardial cells and is widely applied to manipulate gene expression in epicardial cells and epicardium-derived lineages (Simoes and Riley, 2018; Cao et al., 2020). While controversies persist regarding the lineage differentiation of Wt1-CreER labeled epicardial cells into endothelial cells and cardiomyocytes (Zhou et al., 2008; Rudat and Kispert, 2012; Zhou and Pu, 2012; Lupu et al., 2020), there has been a lack of systematic study on Wt1 expression across various cardiac cell types at the single-cell level.

To investigate the function of epicardial cells, we employed a conditional ablation system using *DTA*. *DTA*, a gene-encoded cell toxin, has been extensively utilized to selectively eliminate target cells in various tissues (Sturzu et al., 2015; Fenlon et al., 2020; Xu et al., 2021). In *Rosa26-DTA* mice, DTA expression is controlled by CRE recombinase-mediated gene recombination (Ivanova et al., 2005). Cardiac progenitor cells and cardiomyocytes have been ablated using this system to explore their function in embryonic heart development (Sturzu et al., 2015). The epicardium was selectively ablated in *Wt1-CreER*; *Rosa26-DTA* mice at E11-11.5 to explore its involvement in macrophage recruitment (Stevens et al., 2016). However, a comprehensive examination of epicardium function in myocardium development was not performed.

In this study, we have identified *Krt19* as a novel epicardial cell marker gene. *Krt19*, an intermediate filament protein belonging to the keratin family, functions in maintaining the structural integrity of epithelial cells. Mice homozygous for *Krt19* mutations remain viable and fertile. Notably, *Krt19-CreER* mice have been employed to lineage trace epithelial cells and mesothelial cells across multiple tissues (Means et al., 2008; Westcott et al., 2021). For example, in mouse adult adipose tissue, *Krt19*, but not *Wt1*, was found to be a highly specific marker for the mesothelium (Westcott et al., 2021). Through the combination of single cell RNA sequencing (scRNA-seq), and lineage tracing experiments, we demonstrated that *Krt19* is an epicardial cell gene with differential expression pattern from *Wt1*. We further utilized it together with *Rosa-DTA* mice to study epicardium function.

## Results

### Identification of *Krt19* as a novel epicardial cell marker gene

**FIGURE 1:**
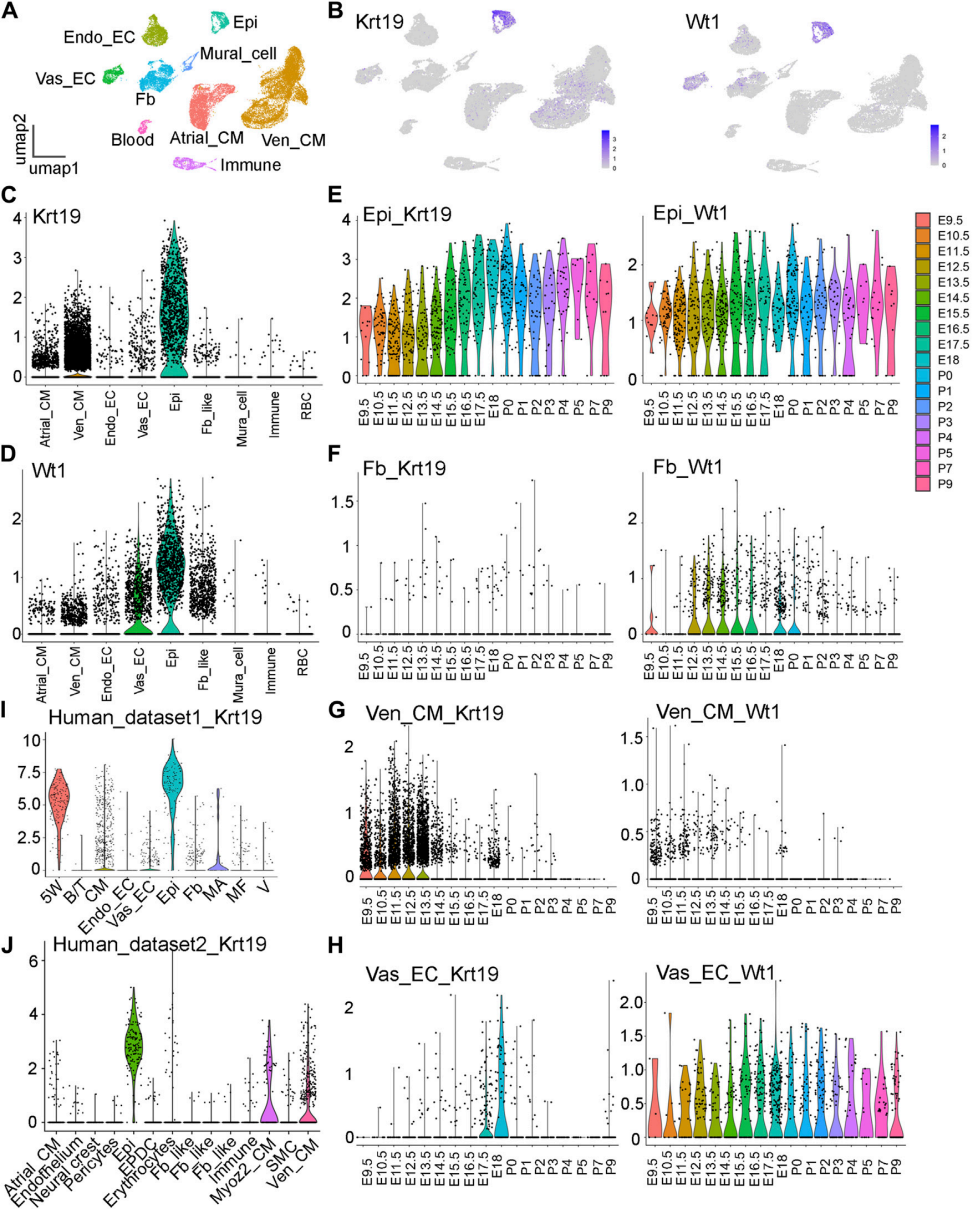
Analysis of the *Krt19* and *Wt1* expression patterns in scRNA-seq data. **(A)** UMAP plot of the CD1 scRNA-seq dataset with cell type annotation. **(B)** Feature plots of *Krt19* and *Wt1* expression in the scRNA-seq data of 18 stages of developing hearts. **(C, D)** Violin plots of *Krt19* and *Wt1* expression at different cardiac cell types. **(E–H)** Violin plots of *Krt19* and *Wt1* expression at different stages in each cell type. **(I, J)** Violin plots of *Krt19* expression at two human fetal heart scRNA-seq datasets.

Through the analysis of an 18-staged cardiac single-cell RNA sequencing (scRNA-seq) dataset in CD1 mice, previously published by our team and deposited in GEO with the accession number GSE193346 (Feng et al., 2022), we identified a new epicardial cell gene named *Krt19* (Figures 1A,B). We found that *Krt19* is highly expressed in epicardial cells at all analyzed stages, ranging from embryonic (E) day 9.5 to postnatal (P) day 9 (Figure 1E). Additionally, *Krt19* is also expressed in atrial and ventricular cardiomyocyte (Atrial_CM, Ven_CM) at early developmental stages (mainly before E14.5) (Figures 1C,G). To better understand *Krt19*’s expression pattern, we compared it with the expression pattern of the well-known epicardial gene *Wt1*. We found that *Wt1* is highly expressed in epicardial cells at all stages (Figures 1B,E) and in atrial and ventricular cardiomyocytes at early stages. However, *Wt1* is also expressed in vascular endothelial cell (Vas_EC) and fibroblast (Fb) at all the analyzed stages, while *Krt19* is barely detected in these 2 cell types at most of the analyzed stages (Figures 1F,H). Moreover, we analyzed *Krt19* expression in two human fetal heart scRNA-seq datasets and found that it had high expression in epicardial cell and moderate expression in CM (Asp et al., 2019; Cui et al., 2019) (Figures 1I,J). In summary, we have identified a novel epicardial cell gene, *Krt19*, which is highly expressed in epicardial cells and exhibits a differential expression pattern compared to *Wt1*.

### Lineage analysis of *Krt19-CreER* labeled cells

**FIGURE 2:**
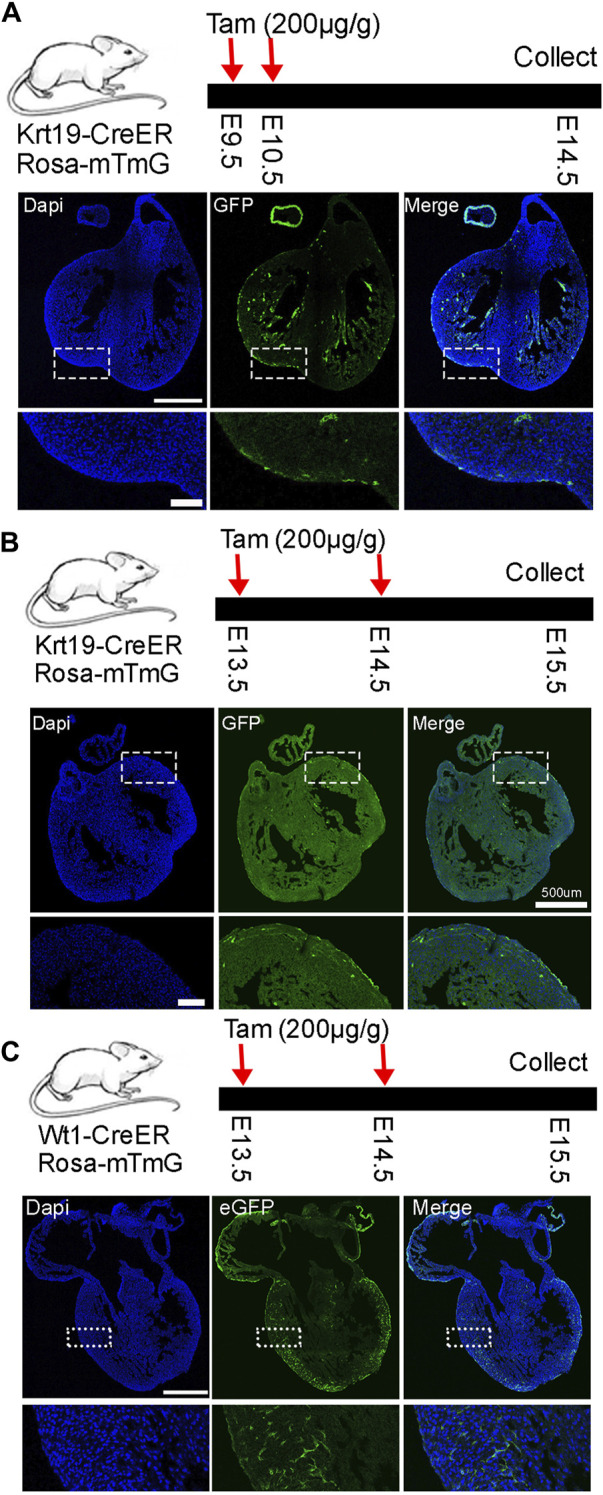
Lineage analysis of *Krt19-CreER* and *Wt1-CreER* labeled cells at early embryonic stages. **(A–C)** Analysis was conducted at early stages by treating the mice with tamoxifen and analyzing them at different stages. Scale bar is 500 µm for the whole heart and 100 µm for the enlarged images.

Next, to understand the lineage development of *Krt19* positive cells, we lineage traced them by breeding *Krt19-CreER* mice with *Rosa26-mTmG* mice and administering tamoxifen at different time points. Initially, we treated the mice with tamoxifen at E9.5 and E10.5 and analyzed their hearts at E14.5 (Figure 2A). We observed strong eGFP signals on the outer surface of the heart, indicating efficient labeling of epicardial cells by the mice. Additionally, we observed eGFP-positive cells inside the chamber and septum, which could be derived from the labeled epicardial cells or non-epicardial cells expressing *Krt19*. We then moved the analysis to a later time window by treating the mice with tamoxifen at E13.5 and E14.5 and analyzing the hearts at E15.5 (Figure 2B). Again, we observed strong eGFP signals in epicardial cells, but also some eGFP-positive cells inside the chamber. In contrast, when we analyzed *Wt1-CreER*; *Rosa26-mTmG* mice at the same time period using the same dose of tamoxifen, we observed strong eGFP signals at the chamber surface and inside the chambers (Figure 2C), which could represent epicardial cells and vascular endothelial cells, respectively. These results are largely consistent with their expression pattern identified in the scRNA-seq analysis.

**FIGURE 3:**
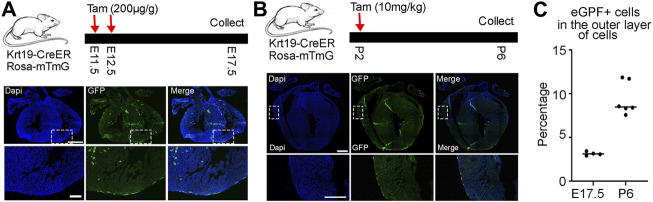
Lineage analysis of *Krt19-CreER* labeled cells at embryonic and neonatal stages. **(A)** Long term lineage analysis of the *Krt19-CreER* positive cells by treating the mice with tamoxifen at E11.5 and E12.5 and analyzing them at E17.5. **(B)** The mice were treated with tamoxifen at P2 and harvested at P6. **(C)** Quantification of the percentages of eGFP-positive cells in the outer layer of epicardial cells. Scale bars are 500 µm for the whole heart and 100 µm for the enlarged images.

Subsequently, we conducted a long-term lineage tracing experiment by treating the mice with tamoxifen at E11.5 and E12.5 and analyzed their hearts at E17.5. We observed clear eGFP signals in epicardial cells and many eGFP-positive cells inside the chamber (Figure 3A). Finally, we treated the mice with tamoxifen at P2 and analyzed their hearts at P6 to understand their performance at the neonatal stage (Figure 3B). We found that all eGFP signals were on the outer surface, indicating that only epicardial cells were labeled by the *Krt19-CreER*; *Rosa-mTmG* mouse line during this time window. Finally, we quantified the percentages of eGFP-labeled cells within the outer layer of epicardial cells. We observed that about 3 percent of cells at the embryonic stage and 9 percent of cells at the neonatal stage were labeled (Figure 3C). However, please note that the labeling efficiency may vary under different doses of tamoxifen treatments.

In summary, the lineage tracing results suggest that the Krt19-CreER mouse line labels epicardial cells at the embryonic stage and displays specificity for epicardial cells at the neonatal stage. This indicates its potential utility in studying the function of epicardial cells and gene regulations within this cell type.

### Functional analysis of *Krt19-CreER* labeled cells at embryonic stage

**FIGURE 4:**
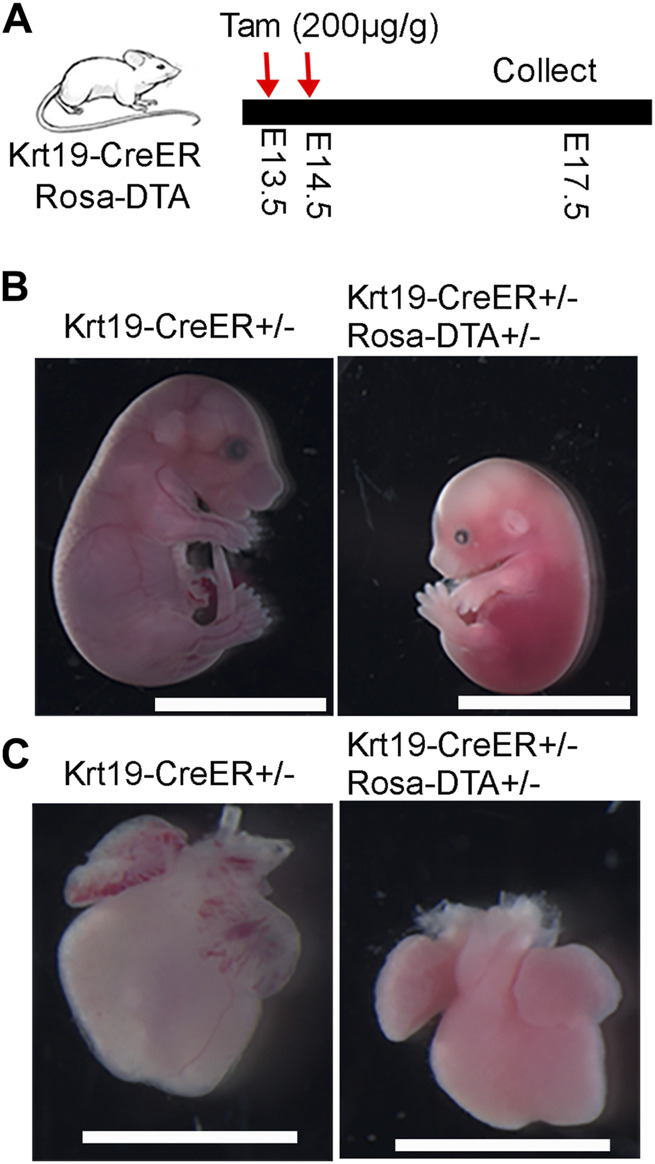
Phenotypic analysis of the embryo and heart defects after ablation of the *Krt19-CreER* labeled cells. **(A)** Diagram of the experimental workflow. **(B, C)** The ablated embryos and hearts were smaller than controls. Scale bar is 1 cm for the embryos and 250 µm for the hearts. Four control and three ablated embryos with similar phenotype were observed in the experiment.

To investigate the function of epicardial cells, we crossed *Krt19-CreER* mice with *Rosa26-DTA* mice for ablation purposes. Tamoxifen was administered to pregnant dams at E13.5 and E14.5, and embryos were collected at E17.5 (Figure 4A). Strikingly, we observed that the ablated embryos, as well as their hearts, were noticeably smaller compared to the controls (Figures 4B,C). These results suggest that *Krt19*-positive cells are essential for embryo and heart development.

**FIGURE 5:**
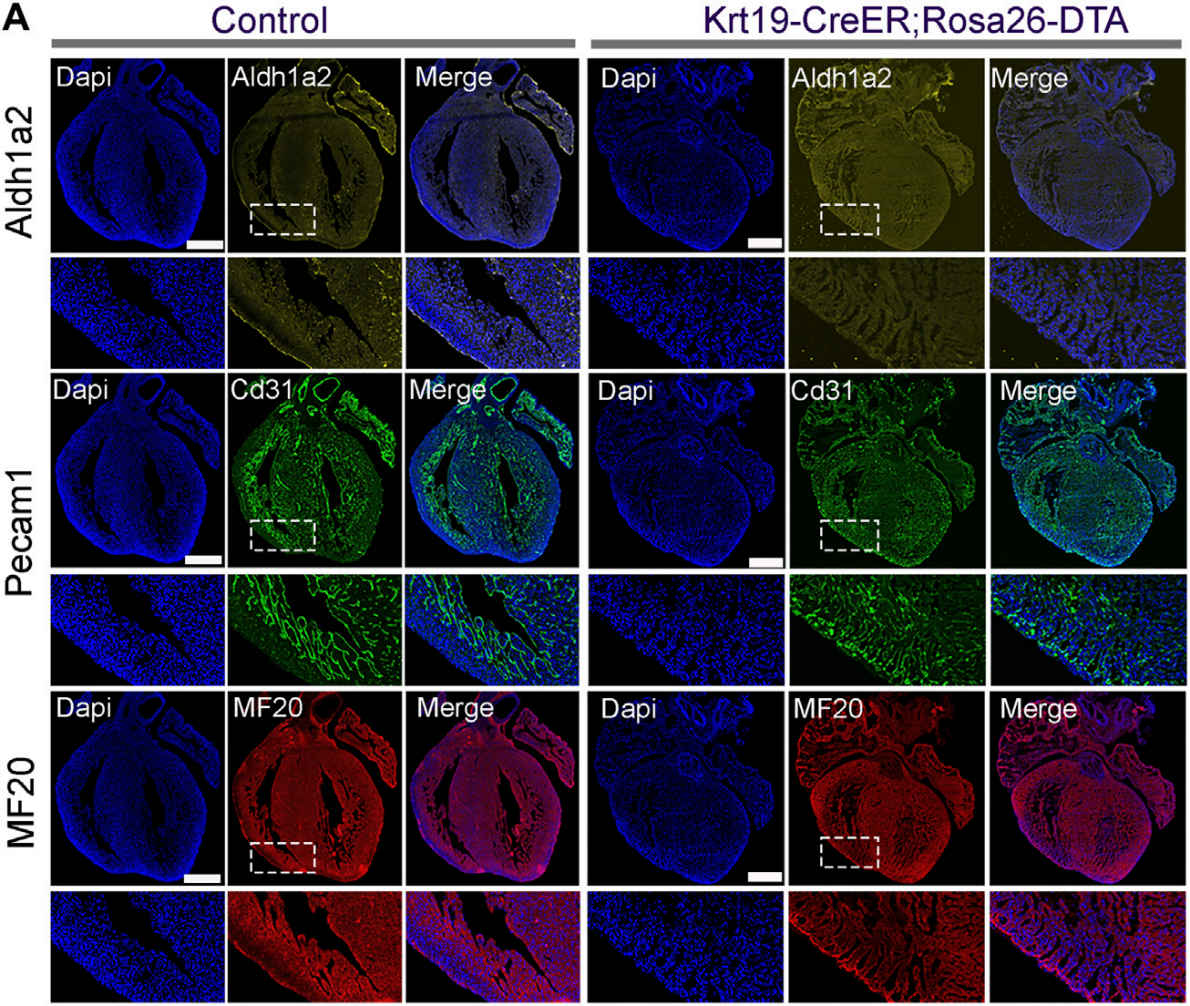
The cellular defects in the ablated embryonic hearts. **(A)** ALDH1A2 signal was largely absent on the outer surface of the ablated hearts. Hyper-trabeculae defects were observed in the ablated hearts based on the staining signal of PECAM1 and MF20. Scale bar is 500 µm in the whole hearts and 100 µm in the enlarged images.

To gain further insights into the molecular defects in the ablated hearts, we conducted immunofluorescence analysis. Firstly, we analyzed the expression of the epicardial cell marker gene *Aldh1a2*. We observed strong fluorescence signals on the outer surface of the chambers in control hearts; however, in the ablation hearts, we found that the signal was largely eliminated (Figure 5A). This result indicates efficient epicardial cell ablation in the *Krt19-CreER*; *Rosa-DTA* mice. Furthermore, we stained the hearts with antibodies against PECAM1 for the endothelial cell lineage and MF20 for the cardiomyocyte lineage. Interestingly, we found that the ablated hearts have an obviously hyper-trabeculated myocardium compared to the control hearts (Figure 5A). These results suggest that the *Krt19-CreER* labeled epicardium is important for embryonic heart development, likely by regulating the growth of compact myocardium.

### The function of *Krt19-CreER* and *Wt1-CreER* labeled cells at neonatal stage

**FIGURE 6:**
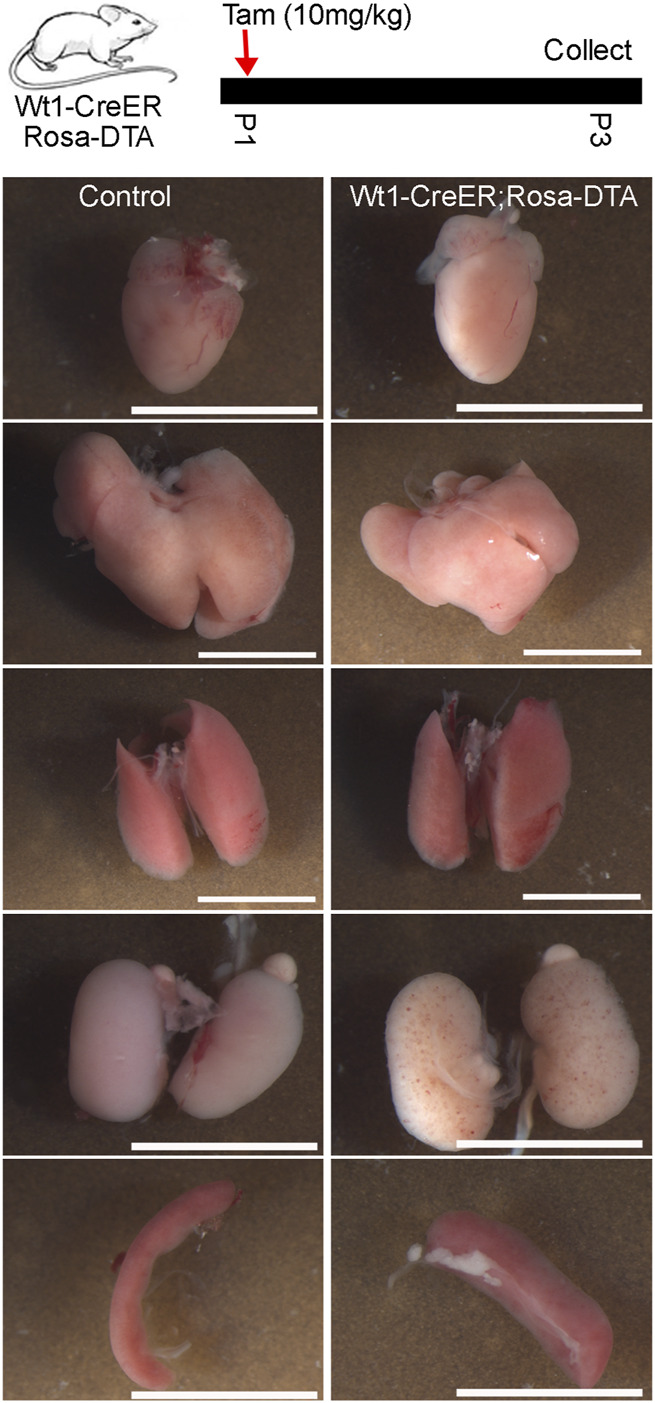
"Multi-organ analysis of control mice and mice subject to Wt1-CreER-mediated cell ablation at the neonatal stage. Tamoxifen was given at P1 and five tissues (heart, liver, lung, kidney, spleen) were analyzed at P3. The scale bar is 5 mm.

Lastly, we crossed *Krt19-CreER* mice with *Rosa-DTA* mice to investigate the function of the epicardium in neonatal mouse heart development. Newborn mice were treated with tamoxifen from P1 to P4, and their hearts were analyzed at P25. We observed that the ablated animal and its heart was noticeably smaller than its controls. Given that we have only successfully retrieved one ablated mouse after multiple breeding, these results can only imply that *Krt19*-positive epicardial cells are likely important for neonatal heart growth (Supplementary Figure S2). Next, we conducted similar experiments with *Wt1-CreER*; *Rosa-DTA* mice, treating them with tamoxifen at P1. However, we found that all the ablated mice died at P4. To better understand the causes of their death, we sacrificed them at P3 for detailed analysis (Figure 6). After inspecting multiple tissues, including the heart, liver, lung, kidney, and spleen from both control and ablated mice, we found that the ablated mice likely died due to kidney hemorrhage or spleen defects, which appeared wider and shorter than those in control mice (Figure 6). These results suggest that utilizing the *Wt1-CreER* mouse line to study epicardium function during neonatal heart development is challenging, while the *Krt19-CreER* mouse line emerges as a potential ideal candidate.

## Discussion

In this study, we have identified a novel epicardial gene, *Krt19*, and found that it exhibits a differential expression pattern from *Wt1*. Furthermore, we conducted lineage tracing experiments to confirm the labeling of epicardial cells by the *Krt19-CreER* mouse line. Finally, we utilized an ablation system controlled by this mouse line to eliminate epicardial cells, thus confirming the importance of the epicardium in embryonic and neonatal heart development.

The epicardium is well known to serve not only as a reservoir of multipotential progenitor cells but also as a crucial source of mitogenic signals orchestrating heart development (Ruiz-Villalba and Perez-Pomares, 2012; Simoes and Riley, 2018; Cao et al., 2020). Anomalies in epicardial development and their signaling mechanisms in diverse mouse models manifest as defective cardiac development, mirroring human CHDs (Ruiz-Villalba and Perez-Pomares, 2012; Gittenberger-de Groot et al., 2016). Notably, the primary CHD arising from aberrant epicardium is left ventricular non-compaction cardiomyopathy, with additional implications for coronary vascular anomalies, valvulopathies, and conduction system anomalies (Zhang et al., 2013; Gittenberger-de Groot et al., 2016). Consequently, undertaking a systematic inquiry into the function of the epicardium becomes imperative, promising valuable insights into the mechanisms underlying CHDs.

Besides epicardial cells, *Krt19* is also expressed in epithelial cells and mesothelial cells in many other tissues, such as adipose and liver (Pepe-Mooney et al., 2019; Westcott et al., 2021). The lethal phenotype observed in *Krt19-CreER*; *Rosa-DTA* embryos could be caused by defects in other tissues. Regarding the defects observed in the hearts, it is possible that they were secondary to defects in other tissues, although the chance is low given the previous publications of a similar phenotype in epicardial gene mutants (von et al., 2011). This issue may also apply to other available epicardium related CreER mouse lines, given that they are also driven by genes expressed not only in epicardial cells but also in other cell types. Furthermore, the defects in the heart’s compact myocardium could potentially be caused by the ablation of *Krt19*-positive CMs. To explore this possibility, we analyzed *Krt19* expressions in compact and trabecular CMs using 18 staged mouse scRNA-seq data. We found that *Krt19* is expressed in both types of CMs and appears to have slightly higher expression in compact CMs (Supplementary Figure S1). However, considering that *Krt19* was mainly expressed in ventricular CMs at stages before E14.5 (Figure 1G), while tamoxifen was administered at E13.5 and E14.5 in the embryonic ablation experiments in Figure 4, and it takes time for the *DTA* gene expression to respond to the tamoxifen treatment, we believe that the embryonic heart defects in *Krt19-CreER*-mediated DTA ablation were likely mainly caused by epicardial cell ablation.

Based on the lineage tracing results (Figure 3B), we have learned that the *Krt19*-CreER mouse line specifically labels epicardial cells at neonatal stage. In contrast, other epicardium labeling strains, including *Wt1-CreER* and *Tbx18-CreER*, have been shown to label epicardial cell and epicardial cell derived cells such as fibroblasts and smooth muscle cells (Cai et al., 2019; Deng et al., 2023). Additionally, the immediate lethal phenotype observed after *Wt1-CreER*-based *DTA* ablation at the neonatal stage also suggests the ablation of critical cell types in other tissues by this line. These results collectively suggest that the *Krt19-CreER* mouse strain may be an ideal model for studying epicardium function and regulation at the neonatal stage. However, Considering that we have only recovered one ablated mouse at P25 from six breedings, including 4 litters of neonatal mice that were not treated with tamoxifen at all (Supplementary Figure S2C), we are cautious about the use of this strain until we understand more about the cause of the low recovery rate. This could be attributed to factors such as the age of the female mice used in the breedings, or potential developmental defects associated with the double heterozygous mice.

## Methods

### Mouse strains

The animal experiments have been approved by the University of Pittsburgh Institutional Animal Care and Use Committee (IACUC). The transgenic mice, including *Krt19-CreERT2* (Strain #:026925) (Means et al., 2008), *Wt1-CreERT2* (Strain #:010912) (Zhou et al., 2008), *Rosa26-mTmG* (Strain #:007676) (Muzumdar et al., 2007), and *ROSA26-eGFP-DTA* (Strain #:032087) (Ivanova et al., 2005) were ordered from the Jackson Laboratory.

### Tamoxifen treatment and mouse dissection

To induce Cre activity, the pregnant mice were given the default dosage of 200 µg of tamoxifen per gram of body weight (200 μg/g) through oral gavage and the neonatal mice were given 10 μg/g of tamoxifen by direct injection into their stomach (Hortells et al., 2020). The pregnant and neonatal mice were euthanized using CO2 and decapitation-based methods, respectively. Following the standard procedure described previously, the mouse hearts were isolated and fixed at 4% paraformaldehyde for immunofluorescence staining.

### Immunofluorescence staining

The staining was performed following a standard procedure. Briefly, mouse hearts were fixed in 4% PFA overnight, embedded in OCT, and sectioned at 10 µm. After a brief wash in PBS to remove the OCT, samples were blocked for 1 h in blocking buffer (10% goat serum, 1% BSA, 0.1% Tween 20) and then incubated with primary antibodies in the primary antibody buffer (1% BSA in PBST) at 4°C overnight. On the second day, the samples were stained at room temperature with fluorophore-conjugated secondary antibodies in blocking buffer for 1 h. Finally, the samples were stained with DAPI, mounted with fluoromount-g, and imaged with a confocal microscope. The antibodies used in the study include anti-CD31 (BD, #550274), anti-Aldh1a2 (Sigma, #HPA010022), and MF20 (DSHB, #MF20). The outer layer of cells in Figure 3C was counted based on the DAPI staining signal.

## Data Availability

The datasets presented in this study can be found in online repositories. The names of the repository/repositories and accession number(s) can be found below: https://www.ncbi.nlm.nih.gov/geo/, GSE193346, GSE106118, and https://ega-archive.org/, EGAS00001003996.

## Supplementary material

SUPPLEMENTARY FIGURE S1 Feature plots showing the expression of compact and trabecular CM genes in Ven_CMs. **(A)** The expression pattern of compact myocardium genes Mycn and Hey2 and trabecular myocardium genes Bmp10 and Slit2. **(B)** The expression pattern of Krt19.

SUPPLEMENTARY FIGURE S2 DTA mediated ablation of Krt19-CreER labeled cells at neonatal stage. **(A)** Diagram of the experimental design. **(B)** Smaller heart was observed after the ablation of Krt19-CreER labeled cells at neonatal stage. Only 1 neonatal heart was analyzed at this stage. **(C)** Summary of the mouse genotyping results from 6 breeding sessions. The scale bar is 5 mm.
