# Supplementary figures and images for: Keratin 19 (Krt19) is a novel marker gene for epicardial cells
Source: Front Genet. 2024 May 20;15:1385867. doi: 10.3389/fgene.2024.1385867 (PMC11145414; doi:10.3389/fgene.2024.1385867)

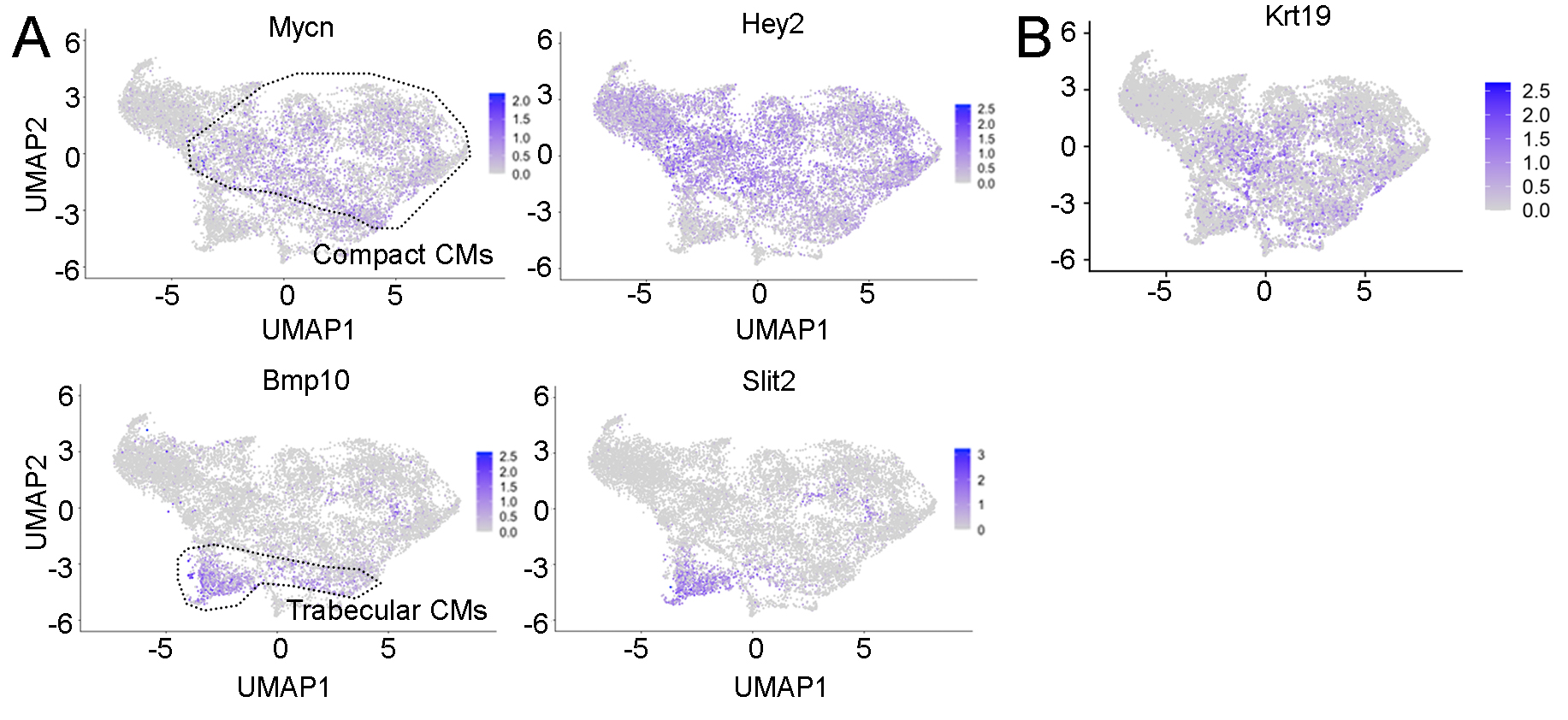

Supplement: Supplementary file 1 [file Image1.jpeg]

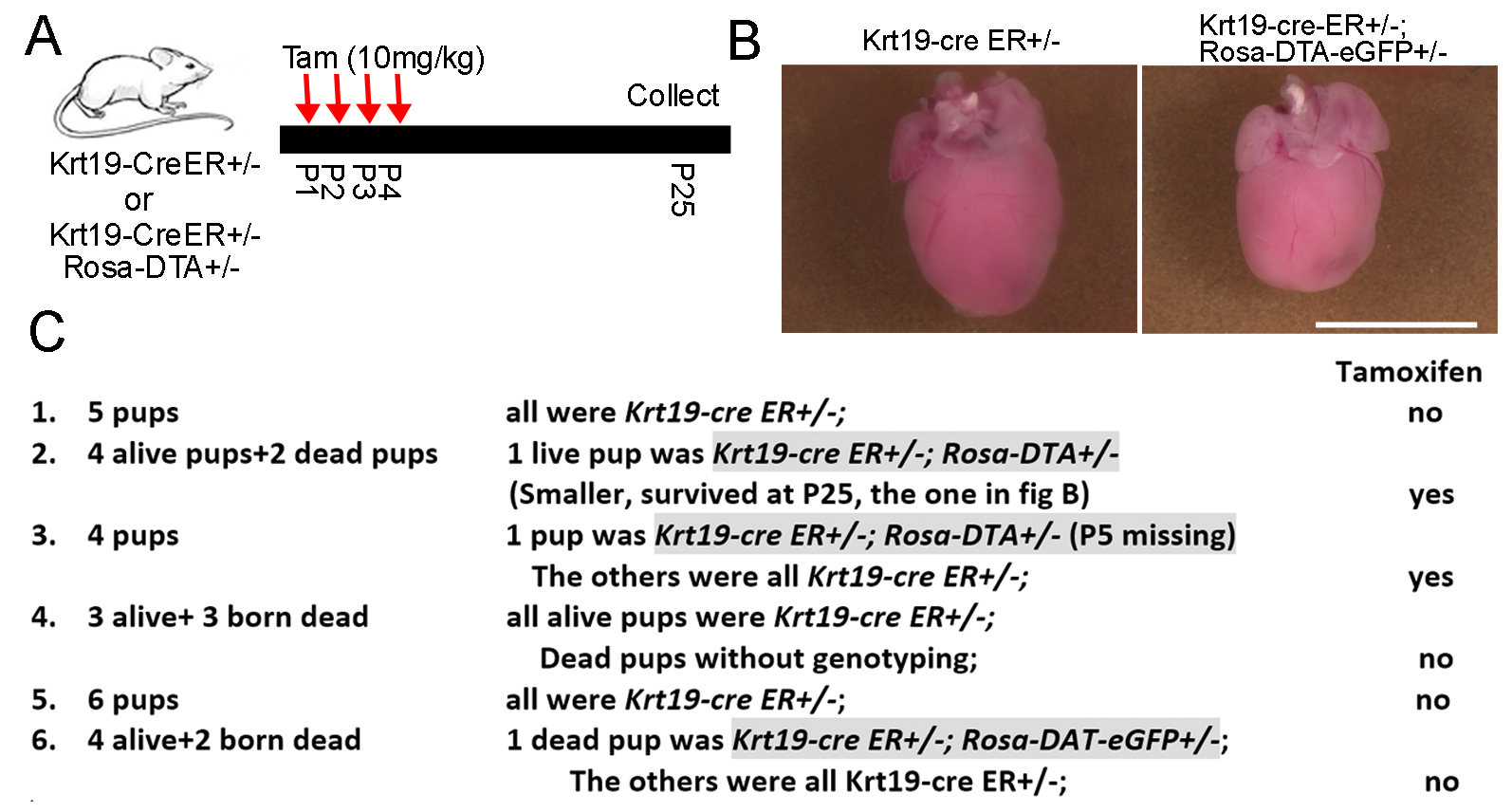

Supplement: Supplementary file 2 [file Image2.jpeg]
